# Prediction of diuretic response to tolvaptan by a simple, readily available spot urine Na/K ratio

**DOI:** 10.1371/journal.pone.0174649

**Published:** 2017-03-31

**Authors:** Yasuyuki Komiyama, Masayuki Kurosaki, Hiroyuki Nakanishi, Yuka Takahashi, Jun Itakura, Yutaka Yasui, Nobuharu Tamaki, Hitomi Takada, Mayu Higuchi, Tomoyuki Gotou, Youhei Kubota, Kenta Takaura, Tsuguru Hayashi, Wann Oh, Mao Okada, Nobuyuki Enomoto, Namiki Izumi

**Affiliations:** 1 Department of Gastroenterology and Hepatology, Musashino Red Cross Hospital, Tokyo, Japan; 2 First Department of Internal Medicine, Faculty of Medicine, University of Yamanashi, Yamanashi, Japan; Taipei Veterans General Hospital, TAIWAN

## Abstract

**Background:**

Tolvaptan is vasopressin type 2 receptor antagonist that inhibits water reabsorption. It is used in combination with standard diuretics to treat ascites unresponsive to standard diuretic therapy or hyponatremia because of liver cirrhosis. This study evaluated the effectiveness and safety of tolvaptan in clinical practice and aimed to determine the factors related to its effectiveness.

**Methods:**

Tolvaptan was administered to 88 consecutive cirrhotic patients with ascites unresponsive to standard diuretic therapy. An effective treatment response was a ≥2% reduction in body weight on day 7. The association of patient pretreatment characteristics with therapeutic effects was analyzed.

**Results:**

Mean weight reduction on day 7 of tolvaptan therapy was −2.9% ± 3.2%, and treatment was effective in 52% of patients. Multivariate analysis revealed that spot urine Na/K ratio ≥2.5 at baseline was the only factor independently related to therapeutic effect, with an odds ratio of 7.85 (95% confidence interval 2.64–23.40, p = 0.0002). Weight reduction percentage on day 7 was −4.0% ± 2.8% in patients with spot urine Na/K ≥2.5, which was significantly greater than the 0.7% ± 2.7% loss in those with urine Na/K < 2.5 (p < 0.05). A spot urine Na/K ratio ≥2.5 had a sensitivity of 85% and specificity of 60% for predicting effective treatment. No adverse events of treatment led to treatment discontinuation.

**Conclusions:**

Baseline spot urine Na/K was predictive of an effective response to tolvaptan therapy. It is simple to perform and readily available and might serve as an indicator of optimal timing of tolvaptan administration in patients with inadequate response to conventional Na diuretic therapy.

## Introduction

Hepatic edema and ascites are common complications of decompensated cirrhosis leading to reduced patient quality of life [1]. Hepatic ascites is refractory in about 10% of patients, and does not respond to salt restriction and standard diuretic drugs [2–5]. Tolvaptan (Otsuka Pharmaceutical, Osaka, Japan) is a nonpeptide antagonist of vasopressin type 2 receptor, a novel class of diuretic drugs. It binds to the vasopressin V2 receptors of principle cells in the renal collecting ducts. It inhibits the expression of aquaporin-2 (AQP2), a vasopressin-regulated water channel, in the apical membrane of renal collecting duct cells. A reduction of AQP2 water channels in the collecting ducts prevents water reabsorption, and promotes urine excretion [6].

Tolvaptan is used to treat patients with heart failure or hyponatremia [7–9], but following successful Phase 3 trials, tolvaptan was approved in September 2013 in Japan for treatment of refractory ascites [10], and is widely used to treat ascites refractory to other diuretics. However, only a few reports of clinical experience have been published [6, 11, 12, 13]. Consequently, patient factors that influence tolvaptan effectiveness are unclear, and the optimal time to begin tolvaptan administration have not been identified.

The guideline of the American Association for the Study of Liver Disease reports a daily urine Na excretion ≥78 mmol/day as an indicator of good ascites control with Na diuretics [3]. It is not easy to measure daily urine Na excretion in routine outpatient care. However, the spot urine Na/K ratio correlates well with daily urinary Na excretion, and can predict responsiveness to diuretics [14–16]. Although spot urine Na/K ratio may be effective as a surrogate marker of daily urine Na excretion, the relationship of spot urine Na/K ratio and tolvaptan therapeutic response is unclear. In this study, we focused on spot urine Na/K and its relationship with tolvaptan effectiveness.

## Patients and methods

### Patients

This prospective observational study included 88 consecutive cirrhotic patients with ascites not controlled by moderate dose of diuretics. All patients received tolvaptan treatment at Musashino Red Cross Hospital between August 2013 and December 2015. Patients with a diagnosis of cirrhosis were eligible if they had ascites unresponsive to standard diuretic therapy consisting of spironolactone, furosemide, or both, in addition to sodium (<5g/day) and water (<1000 mL/day) restriction. The dose of diuretics eligible for inclusion in this was spironolactone≧25mg and/or furosemide ≧20mg. Basically, diuretic therapy was started with spironolactone at dose 25mg or 50mg, then furosemide at dose 20mg or 40mg was added. Addition of tolvaptan was considered if ascites was not controlled by these diuretics. Our policy was to avoid high dose of diuretics because it may cause renal insufficiency. Therefore, tolvaptan therapy was preferentially introduced before the appearance of renal dysfunction. The lack of response to diuretics was defined as the presence of ascites after treatment by furosemide and/or spironolactone for at least 1 months. Patients with overt hepatic encephalopathy, active gastrointestinal bleeding, spontaneous bacterial peritonitis, stage 4 chronic kidney disease (eGFR < 30 ml/min/1.73 m^2^), or heart failure were excluded.

Spironolactone and furosemide dosage was fixed 7 days before, and continued during, tolvaptan administration. Diuretics was not discontinued before or during the study period. Tolvaptan 3.75 mg or 7.5 mg was administered once daily. In the event that the treatment was initiated at a dose of 3.75 mg/day but the response was judged not effective, the dosage was increased to 7.5 mg after few days. In the initial period of this study, we mainly used 3.75mg dose as introduction because we were uncertain of possible adverse events. After confirming the safety of tolvaptan at 7.5mg in several patients, we used 7.5mg dose for most patients. For the baseline data, blood and spot urine test was obtained in the early morning of the day that treatment began, before the administration of diuretics. To avoid hypovolemia and hypernatremia, water intake was not restricted during treatment with tolvaptan in accordance with the instruction of the package insert of tolvaptan. The use of albumin infusion was not restricted during the study period. However, to exclude the short-term effect of albumin infusion on diuretic response, patients who received albumin infusion within 1 week prior to the start of tolvaptan was not included. Patients who received albumin infusion within 7 days after the start of tolvaptan due to insufficient diuretic response to tolvaptan were regarded as ineffective. Patients who had past history of large volume paracentesis were included in the present study. In these patients, tolvaptan was started after we confirmed that patients’ weight became stable after paracentesis.

To date, there is no established criteria to define response to tolvaptan. Therefore, response to tolvaptan was tentatively defined as weight reduction of 2% at day 7 of treatment. Patients with a weight reduction ≥ 2% compared with their baseline weight were recorded as effective cases. Patients who could not continue treatment for 7 days, and patients underwent large volume paracentesis within 7 days after the start of treatment were recorded as ineffective cases. After the patients were stratified into two groups by their response to treatment, the association of demographic and clinical characteristics with the therapeutic effect was analyzed.

### Ethical considerations

This study protocol conformed to the ethical guidelines of the Declaration of Helsinki and was approved by the institutional ethics committees of the Musashino Red Cross Hospital. Written informed consent to receive tolvaptan treatment and to be included in this study was obtained from each study participant.

### Statistical analysis

Statistical analysis was performed with EZR (Saitama Medical Center, Jichi Medical University, Saitama Japan), which is a graphical user interface for R (the R Foundation for Statistical Computing, Vienna, Austria). More precisely, a modified version of the R-commander package with additional statistical functions frequently used in biostatistics [17]. Differences in continuous variables were compared with the paired *t*-test. Differences categorical data values were compared using Fischer’s exact test. Multivariate logistic regression analysis was performed on factors that had a significant relationship with treatment effect in univariate analysis. The cut-off value for continuous variables was determined by receiver operating characteristic (ROC) curve analysis, or by using the reference normal value. A p-value of <0.05 was considered statistically significant.

## Results

### Baseline characteristics of patients

The mean patient age was 69.3 years, and most frequent (53%) underlying liver disease was hepatitis C virus infection. Liver function was Child-Pugh class C in 34 patients (39%); 40 patients (45.5%) had concurrent hepatocellular carcinoma (Table 1). Among 40 patients with HCC, only 1 patient received active treatment for HCC, intra-arterial infusion chemotherapy, during the study period of 7 days. There was no obvious change in the volume of HCC that could cause any improvement of portal hypertension or liver function during the study period. In other patients, active treatment for HCC was not possible because their liver function was too impaired. The median furosemide dose was 20 mg daily; the median spironolactone dose was 50 mg daily.

**Table 1:** Clinical backgrounds of patients.

	n = 88
Age	69.3±12.0
Gender male/female	57(65%)/31(35%)
Child Pugh grade B/C	52(61%)/34(39%)
Etiology of cirrhosis(HCV/HBV/ALD/others)	47/3/22/16
Co-exsisting of HCC	40(45.5%)
Furosemide (mg/day)	20(0–100)
Spironolactone (mg/day)	50(0–100)
Prior history of albumin infusion	22(25%)
Prior history of paracentesis	18(21%)
Albumin (g/dL)	2.6±0.6
Total bilirubin (mg/dL)	1.6±1.1
ALT (IU/L)	32.7±30.2
Creatinin (mg/dL)	1.06±0.81
eGFR (mg/min/1.73m^2^)	65.0±28.6
Serum Na (mEq/L)	136.6±4.8
Platelet counts (×10^4^/μL)	10.0±6.0
Prothrombin activity (%)	71.7±19.3
CRP (mg/dL)	1.14±1.38
Urine osmolarity (mOsm/L)	394±124
Urine Na (mEq/L)	67.6±31.7
Urine K (mEq/L)	23.0±12.2
Urine Na/K	3.67±2.34

Values are mean ± standard deviation or median (range)

HCV: hepatitis C virus, HBV: hepatitis B virus, ALD: alcoholic liver disease, HCC: hepatocellular carcinoma, ALT: alanine aminotransferase

### Treatment efficacy: Weight reduction

The mean weight reduction on day 7 of tolvaptan therapy was 2.9% ± 3.2%. Forty-six patients (52%) achieved a ≥2% reduction on day 7. Symptoms such as abdominal distension or dyspnea was improved in 78% of patients with ≥2% reduction in their weight. Treatment was discontinued before day 7 in two patients who did not respond, but there were no discontinuations caused by adverse events. Seven patients had paracentesis before day 7 and were evaluated with the patients in the ineffective treatment group.

### Treatment efficacy: Correction of hyponatremia

Twenty-two patients had hyponatremia (serum Na < 135 mEq/L) before tolvaptan therapy. There was a trend in increase of serum Na from 130.3 ± 4.4 mEq/L at baseline to 132.2 ± 4.4 mEq/L on day 7 after tolvaptan (p = 0.08). Baseline hyponatremia was corrected (serum Na > 135 mEq/L) in five of 22 patients (23%), but serum Na did not change in patients with normal serum Na at baseline (138.7 ± 2.4 mEq/L at baseline to 138.9 ± 3.3, p = 0.81 on day 7).

### Factors associated with therapeutic effect

**Fig 1:**
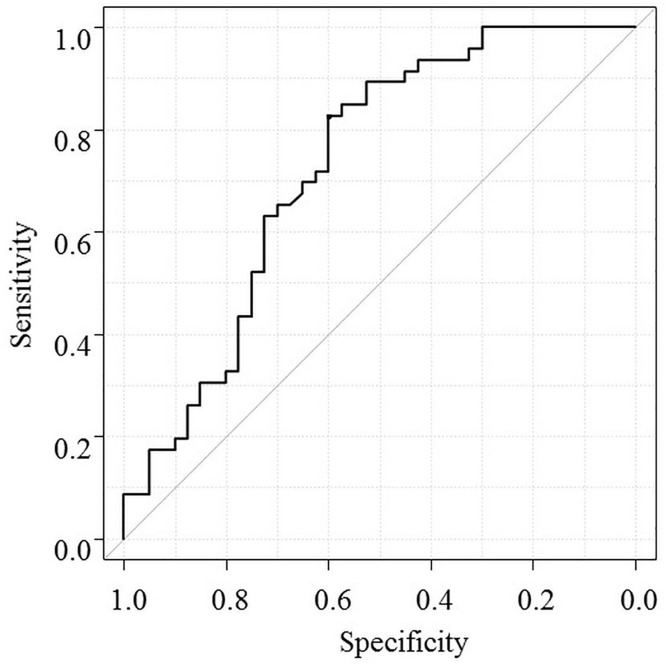
ROC curve analysis of spot urine Na/K ratio and prediction of weight loss. The ROC curve shows baseline spot urine Na/K ratio to predict weight reduction ≥2% on day 7 of tolvaptan therapy. Urine Na/K ratio ≥2.5 was the best cutoff value to predict effectiveness.

**Fig 2:**
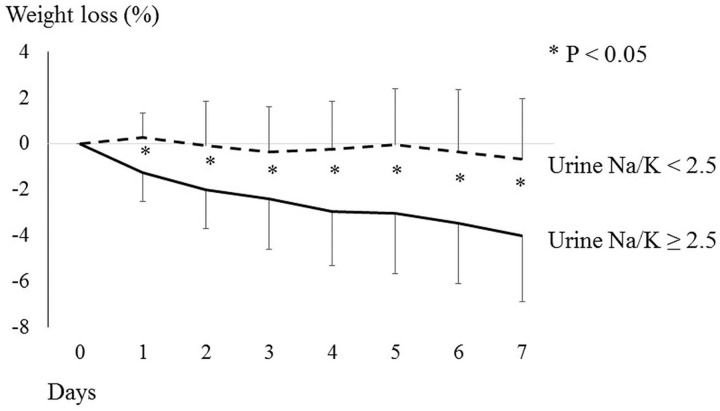
Change in body weight after tolvaptan therapy and baseline spot urine Na/K ratio. The change in mean body weight after tolvaptan therapy is shown over time. The solid line represents patients with baseline spot urine Na/K ≥2.5, and the dotted line represents those with Na/K < 2.5. The error bars indicate standard deviations. Weight loss on day 7 was significantly greater in patients with spot urine Na/K ratios ≥ 2.5 than in those with Na/K < 2.5 (4.0% ± 2.8% vs. −0.7% ± 2.7%, p < 0.05).

Pretreatment characteristics in the effective treatment and ineffective treatment groups were compared (Table 2). There were no significant differences in age, gender, Child-Pugh class, presence or absence of liver cancer, or furosemide/spironolactone dose. When we compare the rate of response between patients with or without prior paracentesis, the rate of response was 33.3% versus 57.1% which was not statistically different (p = 0.11). The effective treatment group had a mean lower C-reactive protein (CRP) level (0.76 ± 0.96 vs. 1.51 ± 1.63 mg/dL, p = 0.01), lower platelet count (8.7 ± 5.4 vs. 11.5 ± 6.3 103/C, p = 0.03), higher mean spot urine Na level (76.5 ± 27.5 vs. 57.3 ± 33.4 mEq/L, p = 0.004), and higher mean spot urine Na/K ratio (4.5 ± 2.2 vs. 2.6 ± 2.0, p < 0.001) than the ineffective treatment group (Table 2). ROC analysis revealed that a spot urine Na/K ratio ≥2.5 was the best cutoff value to determine effectiveness (Fig 1). The prediction accuracy of spot urine Na/K ratio ≥2.5 had sensitivity of 85%, specificity of 60%, positive predictive value of 70%, and negative predictive value of 78%. Multivariate analysis revealed that spot urine Na/K ratio ≥2.5 was the only factor independently related to effectiveness, with an odds ratio of 7.85, 95% CI of 2.64–23.40, and p < 0.05 (Table 3). The percentage weight reduction on day 7 was significantly higher in patients with a spot urine Na/K ratio ≥ 2.5 than in those with a ration <2.5 (4.0% ± 2.8% vs −0.7% ± 2.7%, p < 0.05, Fig 2).

**Table 2:** Baseline factors and response to tolvaptan.

	Effective group Weight reduction ≥2%(n = 46)	Ineffective group Weight reduction <2%(n = 42)	P-value
Age	67.8±13.4	70.9±10.2	0.22
Gender male/female	30/16	27/15	0.99
Child Pugh grade B/C	32/14	26/12	0.5
Etiology (HCV/HBV/ALD/others)	28/0/12/6	19/3/10/10	0.05
Co-exsisting of HCC	17	23	0.13
Furosemide (≦40/ >40mg/day)	37/9	40/2	0.05
Spironolactone (≦50/ >50mg/day)	41/5	42/0	0.06
Albumin (g/dL)	2.6±0.5	2.6±0.6	0.94
Total bilirubin (mg/dL)	1.6±1.0	1.7±1.2	0.7
Serum Creatinin (mg/dL)	0.96±0.64	1.17±0.95	0.22
eGFR (mg/min/1.73m^2^)	70.3±28.2	59.1±28.3	0.07
Serum Na (mEq/L)	137.5±2.9	135.6±6.1	0.06
CRP (mg/dL)	0.76±0.96	1.51±1.63	<0.05
Platelet counts (×10^4^/μL)	8.7±5.4	11.5±6.3	<0.05
Prothrombin activity (%)	72.6±18.2	70.7±20.6	0.66
Urine osmolarity (mOsm/L)	376±110	415±137	0.14
Urine Na (mEq/L)	76.5±27.5	57.3±33.4	<0.05
Urine K (mEq/L)	19.6±9.6	26.8±13.8	<0.05
Urine Na/K	4.5±2.3	2.7±2.0	<0.05

HCV: hepatitis C virus, HBV: hepatitis B virus, ALD: alcoholic liver disease, HCC: hepatocellular carcinoma, ALT: alanine aminotransferase

**Table 3:** Multivariate regression analysis assessing the effectiveness of tolvaptan.

	Odds Ratio (95%CI)	P-value
Platelet counts <15 (×10^4^/μL)	2.51 (0.65–9.65)	0.18
CRP <1.0 (mg/dL)	1.43 (0.49–4.16)	0.50
Urine Na/K ≥2.5	7.85 (2.64–23.40)	0.0002

### Factors associated with urine Na/K ratio

**Fig 3:**
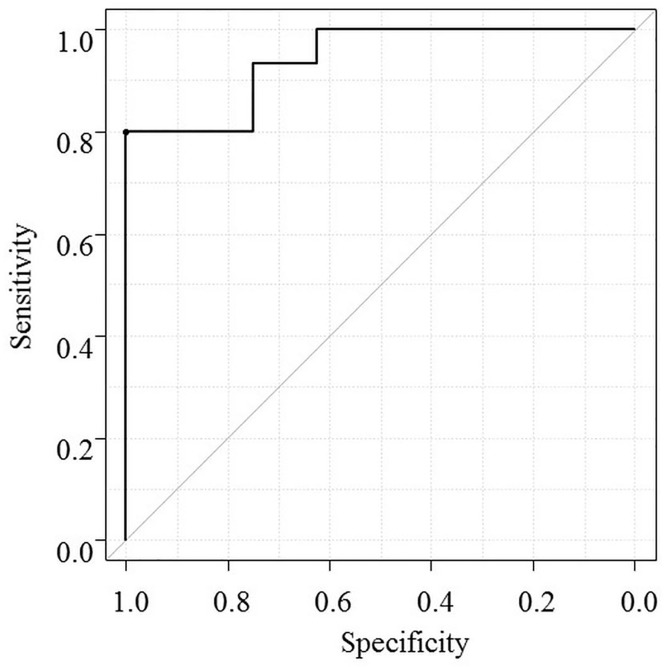
ROC curve analysis of urine Na/K ratio and prediction of total urine Na excretion per day. ROC analysis revealed that urine Na/K ratio was closely associated with urinary Na excretion ≥ 78 mmol/day, with the area under curve of 0.94 (95% CI: 0.85–1.0).

The relationship between spot urine Na/K ratio and total urine Na excretion per day was analyzed in 23 patients with 24-hour urine collection prior to starting tolvaptan. ROC analysis revealed that the spot urine Na/K ratio was closely associated with urine Na excretion ≥78 mmol/day with an area under curve of 0.94 (95% CI: 0.85–1.0, Fig 3). For predicting a urine Na excretion ≥78 mmol/day, spot urine Na/K ratio ≥ 2.5 had sensitivity of 67% and specificity of 100%.

Underlying factors associated with baseline spot urine Na/K ratio ≥2.5 included elevated serum Na levels (138.0 ± 3.3 vs. 134.2 ± 5.9, p < 0.001), low CRP level (0.79 ± 1.02 vs. 1.73 ± 1.71, p = 0.002), elevated urine Na level (169.8 ± 90.7 vs. 54.4 ± 43.5, p < 0.001), and low urinary osmolality (363.6 ± 105.2 vs. 449.8 ± 138.1, p = 0.002, Table 4). Spironolactone or furosemide dose was not associated with spot urine Na/K ratio.

**Table 4:** Urine Na/K ratio and background factors.

		Urine Na/K≥2.5	Urine Na/K<2.5	p-value
		n = 56	n = 32	
Age		68.0±12.2	71.3±11.6	0.22
Gender	male	37 (66.1%)	20 (62.5%)	0.82
	Female	19 (33.9%)	12 (37.5%)	
Etiology of cirrhosis	ALD	15 (26.8%)	7 (21.9%)	0.17
	HBV	0 (0.0%)	3 (9.4%)	
	HCV	31 (55.4%)	16 (50.0%)	
	Others	10 (17.9%)	6 (18.8%)	
Child	B	38 (67.9%)	20 (62.5%)	0.65
	C	18 (32.1%)	12 (37.5%)	
Co-exsisting of HCC		23 (41.1%)	17 (53.1%)	0.37
Furosemide (≦40/ >40mg/day)		47/9	30/2	0.32
Spironolactone (≦50/ >50mg/day)		52/4	31/1	0.64
Albumin (g/dL)		2.5±0.6	2.6±0.4	0.46
Total bilirubin (mg/dL)		1.5±1.0	1.7±1.2	0.34
Serum Creatinine (mg/dL)		1.13±0.97	0.93±0.36	0.26
Serum Na (mEq/L)		138.0±3.3	134.2±5.9	<0.05
eGFR (mg/min/1.73m^2^)		64.2±28.1	66.1±29.9	0.76
CRP (mg/dL)		0.79±1.02	1.73±1.71	<0.05
Urine Na (mEq/L)		82.5±25.4	39.8±22.4	<0.05
Urine K (mEq/L)		18.7±8.6	30.9±14.0	<0.05
Urine osmolarity (mOsm/L)		363±105	449±138	<0.05
Platelet counts (×10^4^/μL)		10.0±6.5	10.1±4.8	0.94
Prothrombin activity (%)		72.9±17.2	69.4±22.6	0.42

HCV: hepatitis C virus, HBV: hepatitis B virus, ALD: alcoholic liver disease, HCC: hepatocellular carcinoma

### Safety

Temporary hypernatremia (serum Na > 145 mEq/L) was observed in four patients on day 2 after tolvaptan introduction, in three patients on day 3, and in one patient on day 7; all patients recovered without discontinuing tolvaptan therapy. Decrease of estimated glomerular filtration rate (eGFR) >25% was observed in six patients in the effective treatment group, and three in the ineffective treatment group. Two patients in the effective treatment group spontaneously improved during the treatment period; three improved after reducing the tolvaptan and furosemide dosages. One patient who did not recover had advanced liver failure caused by exacerbation of the underlying disease. Two of the three patients in the ineffective treatment group had advanced hepatocellular carcinoma, and one had advanced liver cirrhosis with a Child-Pugh score of 12. No patients discontinued treatment because of adverse events, including a patient with liver dysfunction.

## Discussion

In this study, tolvaptan was effective in 52% of patients with ascites not controlled by moderate dose of diuretics such as spironolactone and/or furosemide. A spot urine Na/K ration ≥2.5 prior to administration was predictive of effective tolvaptan treatment. Using this criterion, we were able to include 85% of patients in an effective treatment group. Measurement of spot urine Na/K ratio is easy to do and is readily available, changes during the natural course of liver cirrhosis could be monitored without cost, and thus might be utilized in clinical practice for patient selection or optimize the timing of tolvaptan treatment.

Conventional diuretics used to treat ascites include spironolactone and furosemide which are Na diuretics. Spironolactone is an aldosterone antagonist, and is effective in 50% to 90% of cases [18]. Patients with inadequate responses to spironolactone are given escalating doses of furosemide in combination. However, approximately 10% of hepatic ascites are refractory to these standard diuretic drugs [2–5]; moreover high doses of furosemide has been reported to cause renal dysfunction [19, 20] leading to decreased survival [21].

Tolvaptan, an antagonist of vasopressin type 2 receptor, inhibits water reabsorption and promotes the excretion of free water without increasing Na excretion. Its diuretic mechanism is totally different from conventional diuretics, which promote Na excretion into the urine. Clinical trials in Japan confirmed the efficacy of tolvaptan for refractory ascites regardless of serum albumin level [10], and the safely of a 14-day dosage regimen [22]. Tolvaptan was approved for use in combination with Na diuretics for refractory ascites in 2013. This alternative option for refractory ascites is expected to improve the efficacy and safety of treatment. In the latest version of the Japanese guideline, tolvaptan is recommended to be used in patients who are refractory to diuretics, before considering intra venous administration of diuretics, intra venous administration of albumin, large volume paracentesis, TIPS or peritoneal-venous shunting. In fact, after the approval of tolvaptan, early administration of tolvaptan before increasing furosemide or spironolactone to maximal dose is now common in Japan. Although this agent is widely used in clinical practice, few published reports have described its effectiveness in clinical practice.

Akiyama et al. defined a good patient response as a loss of ≥3 kg body weight loss on day 4 of tolvaptan therapy, and a 46.7% good response rate [12]. Kogiso et al. reported that long-term tolvaptan therapy of 6 months resulted in improvement of the extracellular fluid/total body water ratio in 78.6% of patients [13]. Furthermore, Oki et al. reported that tolvaptan therapy was effective in 63.3% of patients based on improvement in subjective symptoms and a weight loss of ≥2 kg [11]. In this study, 52% of patients experienced a weight loss of ≥2% with 1 week of tolvaptan therapy even though the existing diuretic regimen was not effective. Collectively, tolvaptan was effective in more than half of the patients with refractory ascites, and no patients discontinued treatment because of adverse events. Therefore, we believe that, for refractory hepatic ascites, tolvaptan is an effective treatment option. To date, there is no established criteria to define response to tolvaptan. Therefore, response to tolvaptan was tentatively defined as weight reduction of 2% at day 7 of treatment in the present study. By this tentative definition, symptoms such as abdominal distension or dyspnea was improved in 78% of patients with ≥2% reduction in their weight. Therefore, we believe that this tentative definition of response was clinically relevant.

It would be very helpful to be able to identify those patients who are not likely to respond to tolvaptan in advance of treatment. Currently factors that predict therapeutic efficacy are unclear. Oki et al. reported a hazard ratio of 20.7 for achieving a 25% reduction in urine osmolality when tolvaptan therapy was effective [11]. This finding demonstrates that when tolvaptan is effective to inhibit water reabsorption, it promotes free-water excretion, leading to urine dilution and decrease in osmolality. Nakanishi et al. focused on urine AQP2, demonstrated that urinary AQP2/creatinine decreased after tolvaptan administration, and that the decrease was strongly correlated with decreased urine osmolality [6]. This showed that tolvaptan inhibition of vasopressin V2 receptors can be assessed by urine AQP2. However changes in both urinary osmolality and urine AQP2 occur after tolvaptan administration, thus there are no established pretreatment predictive factors.

In this study, we found that baseline spot urine Na/K ratio was independently associated with tolvaptan effectiveness. As measurement of spot urine Na/K ratio is simple, easily done, and readily available, it can be used in clinical practice to identify patients likely to respond to tolvaptan therapy. As the spot urine Na/K ratio changes during the natural course of liver cirrhosis, it might be used to determine the best time to initiate tolvaptan administration.

Consistent with previous reports, the spot urine Na/K ratio was significantly correlated with daily urinary Na excretion [3, 14, 15, 16]. The association of the spot urine Na/K ratio with tolvaptan efficacy indicates that maintenance of Na excretion was required for tolvaptan effectiveness. This seems reasonable because tolvaptan’s mechanism of action includes inhibition of water reabsorption and promotion of free water excretion without increasing Na excretion. To achieve maximal effect, both Na excretion and free water excretion are necessary.

The present study have some limitations. Patients with TIPS were not included in the present study because TIPS insertion is not performed in our institute. The dose of diuretics was relatively low to moderate, because our policy was to avoid high dose of diuretics to avoid renal insufficiency. Especially, the dosage of spironolactone was particularly low compared to the recommendation by international societies. Although the response to tolvaptan was similar between patients with spironolactone <50mg vs. ≥50mg, it remains unclear whether the similar response to tolvaptan could be achieved by using higher dosage of spironolactone or whether the result of the present study will be applicable in patients receiving a higher dose of diuretics. Therefore, the predictive value of spot urine Na/K ratio for patients under higher dose of diuretics, or those treated by TIPS should be evaluated in the future study.

We conclude that the pretreatment spot urine Na/K ratio can indicate the likelihood of effective tolvaptan treatment, and for ascites patients with insufficient response to conventional Na diuretic treatment, tolvaptan should be introduced when the urine Na/K is ≥2.5 to maximize the efficacy. This simple to perform, readily available criterion could serve to indicate the optimal timing of tolvaptan administration.
